# Novel Single Nucleotide Polymorphisms and Haplotype of *MYF5* Gene Are Associated with Body Measurements and Ultrasound Traits in Grassland Short-Tailed Sheep

**DOI:** 10.3390/genes13030483

**Published:** 2022-03-09

**Authors:** Zhichao Zhang, Cheng Liu, Wenjing Hao, Weiwen Yin, Sitong Ai, Yanfang Zhao, Ziyuan Duan

**Affiliations:** 1 Genetic Resources Center, Institute of Genetics and Developmental Biology, Chinese Academy of Sciences, Beijing 100101, China; zczhang@genetics.ac.cn (Z.Z.); lc244427723@126.com (C.L.); wjhao@genetics.ac.cn (W.H.); yinweiwenahy@126.com (W.Y.); sitongai1995@126.com (S.A.); 2 University of Chinese Academy of Sciences, Beijing 100049, China; 3 Animal Disease Prevention and Control Center, Ewenki Autonomous Banner, Hulunbuir 021000, China; zyfang2008happy@163.com

**Keywords:** *MYF5*, SNPs, haplotype, body measurements traits, ultrasound traits, promoter, sheep

## Abstract

Myogenic factor 5 plays active roles in the regulation of myogenesis. The aim of this study is to expose the genetic variants of the *MYF5* and its association with growth performance and ultrasound traits in grassland short-tailed sheep (GSTS) in China. The combination technique of sequencing and SNaPshot revealed seven SNPs in ovine *MYF5* from 533 adult individuals (male 103 and female 430), four of which are novel ones located at g.6838G > A, g.6989 G > T, g.7117 C > A in the promoter region and g.9471 T > G in the second intron, respectively. Genetic diversity indexes showed the seven SNPs in low or intermediate level, but each of them conformed HWE (*p* > 0.05) in genotypic frequencies. Association analysis indicated that g.6838G > A, g.7117 C > A, g.8371 T > C, g.9471 T > G, and g.10044 C > T had significant effects on growth performance and ultrasound traits. The diplotypes of H1H3 and H2H3 had higher body weight and greater body size, and haplotype H3 had better performance on meat production than the others. In addition, the dual-luciferase reporter assay showed that there are two active regions in the *MYF5* promoter located at −1799~−1197 bp and −514~−241 bp, respectively, but g.6838G > A and g.7117 C > A were out of the region, suggesting these two SNPs influence the phenotype by other pathway. The results suggest that the *MYF5* gene might be applied as a promising candidate of functional genetic marker in GSTS breeding.

## 1. Introduction

The myogenic regulatory factors (MRFs) are essential for regulating the development of muscle fiber and then meat production capacity in mammals [1,2], which include four conserved basic helic-loop-helic (bHLH) molecules functioning as transcription factors as myogenic regulatory factor D (MyoD), myogenic regulatory factor G (MyoG), myogenic factor 5 (MYF5), and myogenic factor 6 (MYF6). All of these four factors affect the determination and maturation of muscle fibers [3]. Variants of these genes can therefore be associated with phenotype of growth, and carcass and meat traits in livestock. Among the four MRFs genes, *MYF5* is the earliest one expressed during embryonic muscle development [4]. Experiments evidently lacking *MYF5* delayed differentiation and modestly impaired proliferation of satellite cells in *MYF5*-null knock-out mice [5], and the fetuses exhibited skeletal defects and died immediately after birth in another experiment [6].

Research about the effect of variants in *MYF5* on animal production traits has been explored in pigs and cattle. In pigs, variation of *MYF5* has been found to be associated with meat quality including loin-eye area, tenderness, total fiber number, water moisture content, and pH value of *longissimus dorsi* [7,8,9,10]. While in cattle, variation in *MYF5* affects growth and meat quality traits including birth, fat, carcass weight, and body length [11,12,13]. In addition, variants of *MYF5* have been reported to be associated with growth and reproductive traits in chicken [14], and showed differences in carcass and meat quality traits in pigeon [15] and rabbit [16].

Ovine *MYF5* maps to chromosome 3 (OAR3) [17]. Currently, there is a report [1] on variants of ovine *MYF5*, which are associated with carcass traits in Romney sheep. No other study exists on ovine *MYF5* describing its variation in performance, especially in body measurements and ultrasound traits.

Given that the association of *MYF5* polymorphism is similar to other livestock in growth and meat quality traits, we hypothesized that there are polymorphisms in *MYF5* in ovine that are associated with the traits of growth and meat quality. The objective of this study was therefore to screen for variations in the promoter, 5′UTR, exons, introns, and 3′UTR regions in the *MYF5* gene of grassland short-tailed sheep (GSTS) in Inner Mongolia, China, and to explore the presence of associations between variants of *MYF5* and growth (body weight and body measurements) and ultrasound traits for revealing its molecular mechanism preliminarily.

## 2. Materials and Methods

### 2.1. Experimental Animals

A total of 533 adult GSTS (430 ewes and 103 rams) in Inner Mongolia, China were randomly selected from their breeding farm in Ewenki Autonomous Banner, Inner Mongolia Autonomous Region. These sheep were grazed in the steppe all year round, moved freely, and were in good health.

### 2.2. Data Collection and Sampling

The blood samples were collected from the jugular vein of sheep (5 mL/sheep) by a qualified veterinarian and stored in blood collection vessels containing EDTA. All operations followed the guideline of Ai Yi Ti Breeding farm of grassland short-tailed sheep, Inner Mongolia Autonomous Region (approval number: AYT-2020112003). Genomic DNA was extracted using the TIANamp Genomic DNA Kit (TIANGEN, Beijing, China) following the manufacturer’s instruction. The DNA purity was tested by Spectrophotometer and dissolved in TE buffer (10 mM Tris-HCl, 1 mM EDTA, pH 8.0), and stored at −20 °C.

Growth traits including body weight (BW), body length (BL), withers height (WH), chest depth (CD), chest circumference (CC), chest width (CW), cannon bone circumference (CBC), and hip width (HW) were determined as is routine method. Ultrasound traits including eye muscle area (EMA) and backfat thickness (BFT) were measured by the animal specific B ultrasonic instrument whilst the animals were alive (Dawei, Xuzhou, China). Animals to be tested were restrained in a standing position after shaving of the wool and cleaning of the skin on the right side of the region between the 12th and 13th thoracic vertebrae; the ultrasound images of the longissimus muscle were captured using the procedure described by Rozanski et al. [18]. At the end of the measuring, all images of each individual were analyzed by the same person who operated the ultrasound apparatus, using the software fixed in the instrument in order to eliminate systematical error.

### 2.3. Identifying SNPs and Genotyping

According to the ovine *MYF5* sequence provided in Genbank in NCBI (Ovis aries. NC_040254.1), seven pairs of sequential overlapping primers were designed using Primer Premier 5, each pair of primers can amplify about 800 bp in length, and are named MYF5-1, MYF5-2, and MYF5-3, etc., in numerical order, covering the open reading frame (ORF), promoter, 5’UTR, and 3’UTR. The information of the primers is shown in Supplementary Materials Table S1.

For SNPs detection, twenty genomic DNA samples were selected randomly as a pool to identify if mutation of the gene was harbored. PCR was performed in a 25 μL reaction volume, including 2 × Taq PCR MasterMix (12.5 μL), forward and reverse primer (10 μM, 1 μL for each), DNA (1 μL), and ddH_2_O (9.5 μL). The reaction of thermal cycling included: 95 °C for 5 min, followed by 35 cycles: 95 °C for 30 s, 60 °C for 40 s, and 72 °C for 1 min; and 72 °C for 7 min for the final extension, then the samples were stored at 4 °C. Bidirectional sequencing was operated by Sangon (Shanghai, China). The Chromas software was employed to analyze and determine the location of the SNPs.

After sequencing detection, the technique of SNaPshot was used to type each specific location of SNPs found and then the other seven primers were designed according to the description of Turner et al. (2002), labeled as SNP1, SNP2, and SNP3 etc., in numerical order. The information about the primer pairs is shown in Supplementary Materials Table S2. The PCR reaction mixture was 25.0 μL, including 1.1 × T3 Super PCR Mix 22 μL (TSINGKE, Beijing, China), 10 μM primer F 1 μL, 10 μM Primer R 1 μL, and gDNA 1 μL. The thermal cycling procedure was 98 °C for 2 min, followed by 35 cycles at 98 °C for 10 s, 60 °C for 10 s, and 72 °C for 20 s; then 72 °C for 5 min, and the products were stored in 4 °C.

### 2.4. The Determination of Transcriptional Regulation of SNPs in the Promoter Region

#### 2.4.1. Cell Culture

The 293T cell line (HEK293T) was purchased from the American Type Culture Collection (ATCC, Manassas, VA, USA), and the cells were cultured in Dulbecco’s modified Eagle medium (DMEM) with 10% fetal bovine serum (FBS) (Ioway, Beijing, China) and 1% penicillin and streptomycin (Invitrogen, Carlsbad, CA, USA). Sheep embryonic fibroblasts cell line (SEF) was courtesy of Professor Ma RZ from the University of Chinese Academy of Science, and the selection of the sheep embryonic fibroblasts was conducted with DMEM/F12 medium. Both of the cells were inoculated into 24-well plates at the density of 1 × 10^5^ cells/well and cultured in 5% CO_2_ incubator at 37 °C. Cells were treated with 0.1 μM Plicamycin as SP1 inhibitor (Cat#HY-A0122, MedChemExpress, Monmouth Junction, NJ, USA) followed by transfection for 6 h.

#### 2.4.2. Construction of Dual-Luciferase Reporter Expression Vectors

According to CE Design of Vazyme primer software (http://www.vazyme.com, accessed on 22 March 2021), five fragment primers starting in +59 bp with successive deletions of −1799, −1197, −866, −514, and −241 in the promoter region of *MYF5* were designed (the primers’ information is shown in Supplementary Materials Table S3), and double restriction sites of XhoIand HandIII were added at each ends of the fragment as PGL4.2-Basic vector request, which were named as PGL4.2-1799, PGL4.2-1197, PGL4.2-866, PGL4.2-514, and PGL4.2-241, respectively. Each insertion of fragments with the same 25 base pairs at 5′ and 3′ ends corresponding to the PGL4.2-Basic was homologously recombined using Clon Express^®^ Ultra One Step Cloning Kit (Vazyme, Nanjing, China), and the ligated products were transformed into DH5α competent cells.

#### 2.4.3. Dual-Luciferase Reporter Assay

The 293. T and SEF cells were inoculated into a 24-well plate at a density of 1 × 10^5^ cells per well initially, and cultured in a 5% CO_2_ incubator at 37 °C. The cells were transfected when the fusion reached approximately 80% within 24 h. A total of six recombinant plasmids (including one empty plasmid) with 1000 ng and TK-Renilla plasmid with 15 ng were co-transfected into 293T and SEF cells, and each of experiments were repeated three times. After 48 h transfection the cells were collected, and the firefly luciferase (F) and sea kidney luciferase activity (R) were determined by the Dual-Luciferase Reporter^®^ Assay System (Promega, Madison, WI, USA) and detected by GloMax 20/20 Luminometer (Promega, Madison, WI, USA). The firefly luciferase activity levels in each well were normalized by those for Renilla luciferase. All of the experiments were replicated three times and each assay was performed thrice.

#### 2.4.4. Prediction of Transcription Factors in the Promoter Region

Alibaba 2.1 (http://gene-regulation.com/pub/programs/alibaba2/index. Html, accessed on 13 July 2021) online tool was paired with the input complete sequence of *MYF5* containing SNP1, 2, and 3 from NCBI firstly, the specific parameters set as: the predicted length is 50 bp, the longest binding site in length is 10 bp, and the shortest is 4 bp, with the confidence above 80%. Then, the predicted transcription factors were further analyzed on the JASPER website (http://jaspar.genereg.net/, accessed on 13 July 2021), both with highest scores were selected.

#### 2.4.5. Construction Luciferase Reporter Plasmid with Mutation

To investigate the difference of luciferase activity among different genotypes of SNP2, a 48 bp (−695 to −742 bp) wild-type sequence containing SNP2 was inserted into PGL4.2-Basic as PGL4.2-SNP2-W, then the site G in wild-type was mutated into T by PCR method to construct a mutant plasmid as PGL4.2-SNP2-M. The constructed plasmid was verified by double digestion and Sanger sequencing. A blank control and the experimental groups were tested for three times in SEF cells. The information of the base mutation primers is shown in Supplementary Materials Table S4.

### 2.5. Statistical Analysis

The genotype and allele frequency were calculated directly. The polymorphism information content (PIC) was used to estimate the *MYF5* in population.

(1) $$\mathrm{PIC}=1-\sum_{i=1}^{m}p_{i}^{2}-\sum_{i=1}^{m-1}\sum_{j=i+1}^{m}2p_{i}^{2}p_{j}^{2}.$$

$p_{i}$ and $p_{j}$ are the frequencies of i and j alleles, respectively, and *n* is the number of multiple alleles. PIC > 0.5 indicates that the locus is highly polymorphic, when PIC < 0.25 means the locus is in low polymorphism condition, and 0.25 < PIC < 0.5 suggests that the locus is moderately polymorphic [19].

The Hardy–Weinberg equilibrium (HWE) was evaluated by the Chi-squared test in Haploview 4.2 (Whitehead Institute for Biomedical Research, Cambridge, MA, USA). The r^2^ and D’ linkage disequilibrium (LD) and haplotype analysis were also calculated with Haploview 4.2.

In this experiment, the general linear model (GLM) as Y*_ij_* = μ + G*_i_* + S*_j_* + e*_ij_* was used for analyzing the association between SNPs and phenotypes by SAS 9.4 (SAS Institute Inc., Cary, NC, USA), where Y*_ij_* was the phenotypic observations; μ was the averaged values; G*_i_* was the fixed effect of genotype; S*_j_* was the fixed effect of sex; and e*_ij_* was the residual effect. All values were expressed as the mean ± standard deviation. The results with *p* < 0.05 were considered statistically significant.

## 3. Results

### 3.1. SNP Detection and Genetic Parameters

**Figure 1:**
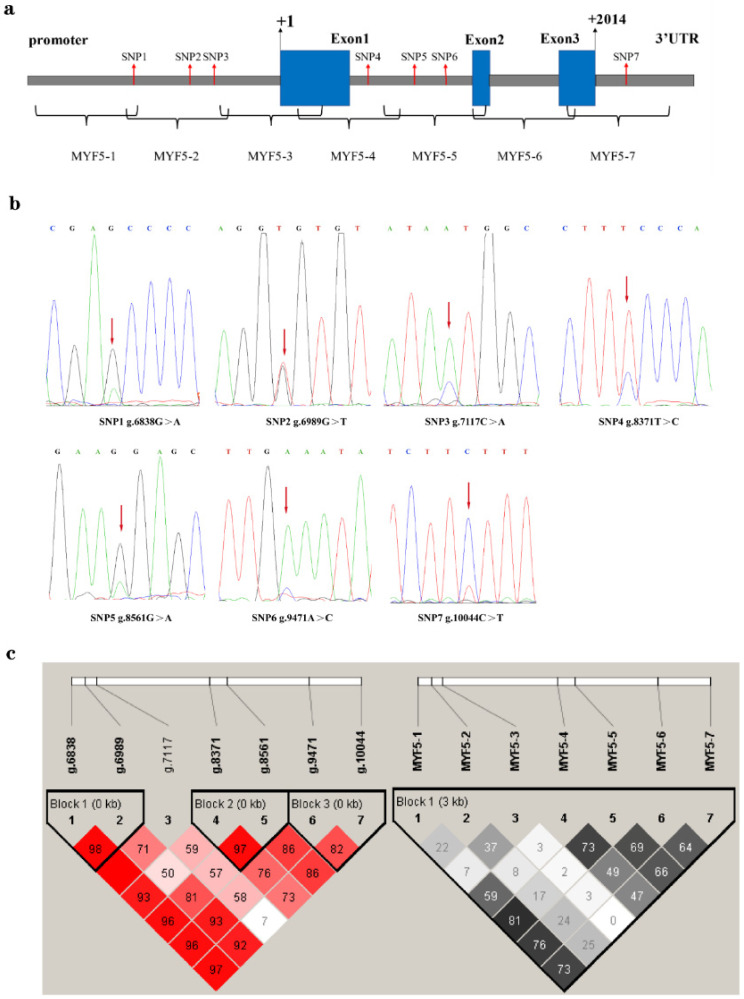
Structure of the *MYF5* gene (**a**); Sequence variants of the *MYF5* gene in GSTS (**b**); Linkage disequilibrium coefficients (D’ left, R^2^ right) among *MYF5* SNPs (**c**).

A total of 7 SNPs have been detected in this study by direct sequencing the pool PCR products from seven sequential primers for *MYF5* (Figure 1), SNP1, SNP2, and SNP3 locate in the promoter, SNP4 and SNP5 in the first intron, SNP6 in the second intron and SNP7 in the 3′UTR region of the gene; the detailed information is shown in Table 1 and Figure 1. Based on the information above, 533 samples were genotyped by SNaPshot technology. Genetic parameters of genotype and allele frequency, observed heterozygosity (ObsHe), predicted heterozygosity (PredHe), χ^2^ test for Hardy–Weinberg equilibrium (HWE), and the polymorphism information content (PIC) were calculated for all 7 SNPs and shown in Table 1. All SNPs were in Hardy–Weinberg equilibrium and the genetic diversity of SNP1, SNP5, SNP6, and SNP7 were in low polymorphism condition (PIC < 0.25), and SNP2, SNP3, and SNP4 were in moderate status (0.25 < PIC < 0.5).

### 3.2. Linkage Disequilibrium and Haplotype of the SNPs

The linkage disequilibrium coefficients of *D’* and *r^2^* values (*D’* = 1 indicates full linkage, *r^2^* > 0.33 indicates strong linkage) showed that between SNP4 and SNP5 (*D’* = 0.97, *r^2^* = 0.73), SNP6 and SNP7 (*D’* = 0.82, *r^2^* = 0.64) and SNP1 and SNP2 (*D’* = 0.98, *r^2^* = 0.22) were in strong linkages states, and the degree of linkage went from high to low. As Figure 1c displayed, these 7 SNPs formed three haplotype modules in STGS flock.

The haplotype analysis indicated there were eight haplotypes with the frequencies more than 1% in the sampled sheep flock, and H1(-GTATGTC-) was the most abundance one with the frequency of 49.7%, followed by H2 (-GGCTGTC-) and H3 (-AGACAGT-) with 22.2% and 12.6%, respectively (Table 2). Based on the pairwise combination of the eight haplotypes, four major combinations of diplotypes with the frequencies above 5% were identified, which account for 66.3% of the sampled sheep covering 373 individuals, and the other combinations were too small to be considered. Among four combinations, H1H1 was the most common one (GTATGTC/GTATGTC, *n* = 144, 25.6%) in the tested flock, followed by H1H2 (GTATGTC/GGCTGTC, *n* = 128, 22.4%), H1H3 (GTATGTC/AGACAGT, *n* = 59, 10.5%), and H2H3 (GGCTGTC/AGACAGT, *n* = 42, 7.5%) (Table 2 and Table 3).

### 3.3. Association of the SNPs and Haplotype Combinations with Phenotype Traits

Through genotyping, three mutations were determined as SNP1, SNP2, and SNP3, which existed in the promoter region. For the SNP1, the sheep with AA genotype has significantly higher BW, WH, CC, and SC than GA and GG ones (*p* < 0.05). Moreover, individuals of AA genotype had significantly higher CW than that of GG genotype (*p* < 0.05). For SNP3, the EMA of AC genotype individuals was significantly higher than that of CC (*p* < 0.05), while there was no difference between CC and AA (*p* > 0.05). However, there was no significant association detected between the mutants and the phenotypes for SNP2 (Table 4).

The other SNPs, namely SNP4, SNP5, and SNP6, are located in the intron regions of *MYF5* and had complicated association with phenotype traits. SNP4 is located in the first intron, and the heterozygous genotype CT had more advantages in BW, BL, WH, CC, CBC, and EMA than CC genotypes (*p* < 0.05). TT genotype also had higher mean values of BW, CBC, and EMA than CC genotype. Meanwhile, CC, CT genotype had significantly higher WH than CC and TT genotypes (*p* < 0.05), but there was no significant difference between CC and TT genotypes (*p* > 0.05).

SNP5 was also located in the first intron, but there is no significant difference among genotypes (*p* > 0.05). For SNP6, it was located in the second intron of *MYF5* gene, and the individuals with GG genotype had significantly higher values in BW, WH, and CBC than individuals with TT genotype (*p* < 0.05). Genotype GT was in moderate level in all phenotype traits, except that CBC was significantly lower than GG genotype.

For the 3′UTR mutation of SNP7, the sheep with TT genotype had higher BW and greater WH than CT and CC genotype (*p* < 0.05). Besides, TT genotype had significant higher mean values of chest circumference (CC) than CC genotype, and there was no difference (*p* > 0.05) between CT and CC genotype.

The result of association analysis amongst the four diplotypes and phenotypic traits showed that the mean value of BW, BL, WH, CC, CW, CBC, and HW of H1H3 and H2H3 were significantly (*p* < 0.05) higher than those of H1H1 and H1H2. However, there were no significant differences (*p* > 0.05) among the four diplotypes in two ultrasound traits (EMA and BFT) (Table 5).

### 3.4. An Optimal Haplotype for Meat Production Breeding

According to the results above, it seems that individuals with haplotype H3 had a better performance than the others, so all individuals were divided into two groups, which were animals with H3 haplotype (H3+) and animals without H3 haplotype (H3−). The result of contrastive analysis showed that H3+ group had significant (*p* < 0.05) advantages over H3- group in BW, BL, WH, and CW (Table 6).

### 3.5. The Position and Binding Factor of Three SNPs Functioned in Ovine MYF5 Promoter

**Figure 2:**
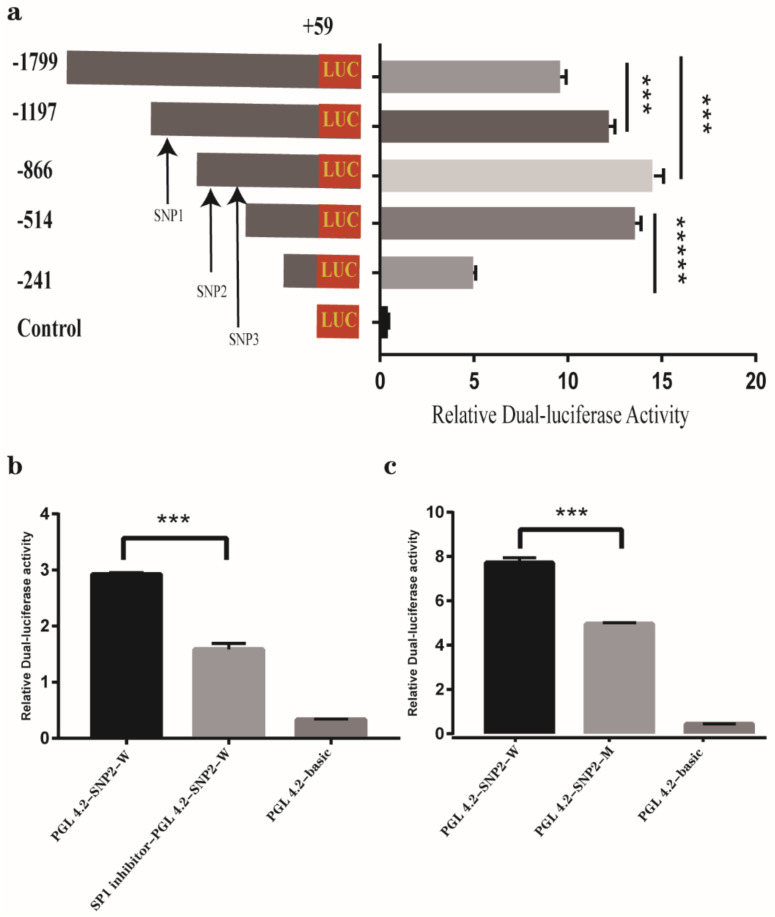
Promoter activity analysis of ovine *MYF5* gene in sheep embryonic fibroblast (**a**); The effect of SP1 inhibitor to the PGL 4.2-SNP2-W plasmid (**b**); Comparison of the relative dual-luciferase activity between wild-type and mutant PGL4.2-SNP2 plasmid (**c**). Results are shown as mean ± standard deviation and the data are representative of at least three independent assays. The statistically significant difference between groups was tested by independent sample *t*-test. *** *p* < 10^−3^, ***** *p* < 10^−5^.

After successfully co-transfecting the plasmids of successive truncating fragments of ovine *MYF5* promoter with Renilla plasmids into 293T or SEF cells in 24 h, the dual-luciferase activity detecting results tended to be consistent in the two cell lines (only the result of SEF is shown), which indicated that all five truncation fragments are in active, and the fragment from −514 to −241 bp showed the highest activity (*p* < 10^−5^) (Figure 2a); this indicated, therefore, that the functional core region of ovine *MYF5* gene promoter is located between −514 to −241 bp. However, it is a little surprising that SNP1, 2, and 3 are out of the core region. Certainly, the fragment containing SNP 1, 2, and 3 (−1799 to −866 bp) also had a significant effect (*p* < 0.001) with the promoter activity (Figure 2a). It is logical to assume that these three SNPs play an important role in regulating the promoter activity.

The results of prediction for the binding sites to transcription factor on the three SNPs showed that SP1 is located in the binding site of SNP2, and there are no binding sites on SNP1 and 3. Hence, the SP1 inhibitor assay conducted, and the result displayed that the luciferase activity decreased significantly (*p* < 0.001) when SP1 inhibitor added to the culture of SEF cells transfected with PGL4.2-SNP2-W plasmid (Figure 2b). Further, the mutant of PGL4.2-SNP2-M plasmid was transfected, and the activity was significantly (*p* < 0.001) lower compared to the wild-type (Figure 2c). This suggests that the binding of SP1 to SNP2 plays an important role in exerting the promoter activity of *MYF5*.

## 4. Discussion

There were few studies on variations in ovine *MYF5,* especially the variations associated with the production traits in sheep. Sousa et al. [20] had genotyped *MyoD* family genes including *MYF5* in 192 Santa Inês sheep and also analyzed association with meat quality traits, and had not found an association with *MYF5*. Phua and Wood [21] firstly reported two variations in ovine *MYF5* in 24 sheep samples using Southern hybridization RFLP method. Wang et al. [1] detected two SNPs in exon 3 and an indel in intron 2 from five breeds with total of 645 sheep samples using PCR-SSCP analysis, and found genotype AA from Romney sheep associated with carcass lean meat yield. More *MYF5* sequence variation has been described in cattle [12,13,22] and pigs [10,23,24].

In the study, the ovine *MYF5* gene was genotyped with seven primers spanning 4606 bp within an indigenous Chinese breed named GSTS and seven SNPs were determined from 533 individuals located in the promoter (SNP1, SNP2, and SNP3), intron 1 (SNP4 and SNP5), intron 2 (SNP6), and 3′UTR (SNP7) of *MYF5*, respectively. Among them, except for three SNPs in the intron 1 and 3′UTR, which were reported previously in Santa Inês sheep [20], the SNP1, SNP2, SNP3, and SNP6 were detected firstly in Ovis aries. Except for SNP2, this study also revealed significant association (*p* < 0.05) between SNPs, body measurements, and ultrasound traits in a relatively large amount of sheep samples. This might be the reason that previous studies failed to find the variations in ovine *MYF5* gene and the association with measurements of phenotype. The results above further confirmed that *MYF5* gene had an effect on the body measurements traits of domestic animals.

Usually, a single SNP has little effect on a phenotype, while haplotype could provide more abundant information, because haplotype composite heterozygous SNPs are located on single chromosomes, play an important role in the mining of disease genes, and search for new strategies for disease treatment [25]. In the present study, we detected eight haplotypes consisting of seven mutant sites with a frequency above 1%, and the most common haplotype is H1 (0.497) in the GSTS population. Through analyzing the association between different haplotypes with the body weight, body size, and ultrasound traits in GSTS flock, we also found that haplotype H3 had a better performance in body weight, body size, and ultrasound traits, hence the combinations of H1H3 and H2H3 showed a higher mean value than other major diplotypes, suggesting that H3 was a beneficial haplotype for body measurements and carcass traits, which laid the foundation for formulating the breeding regime. It is anticipated that strengthening the selection of H3 haplotype would increase the frequency of H3 in the population of the sheep flock, resulting in desirable body measurements traits for improving the meat production performance at an accelerated rate for GSTS.

In the study, seven mutants of SNP were detected in the flock, but no SNPs were found in exon, suggesting that the exon region of *MYF5* in GSTS tends to be relatively conserved. It was believed that mutations occurring in intron could not affect biological traits. With the progressing of research, it is found that introns are involved in lots of gene regulation such as splicing in eukaryotes. The variants in introns may lead to change splicing accuracy or efficiency, resulting in changes in amino acid coding and ultimately change in the phenotype of eukaryotes [26]. For example, the variation in the third intron of the *IGF2* may affect its binding to the transcription factor ZBED6, which would then improve the meat yield of Chinese Bama pigs [27]. In the study, we detected three SNPs in the intron regions of ovine *MYF5*, and CC homozygous genotype of the SNP4 had a negative effect on body weight, body measurement traits, and eye muscle area, but CT heterozygous had the highest mean value in all measuring traits among the three genotypes. Comparatively, the mutation of SNP6 had a positive effect on body weight, withers height, and cannon bone circumference. These results supplied clues for further investigation in the mechanisms of transcriptional regulation in gene introns.

Generally, the region of 3′UTR involved the binding site of mRNA and therefore regulated nuclear transport, polyadenylation, and subcellular targeting, as well as rates of localization, translation, and mRNA-specific degradation [28,29]. The function of miRNAs appears to be negative regulators for transcription by interacting with 3′UTR of target gene [30]. Thus, sequence alterations in the region of 3′UTR may involve an inappropriate expression of genes and has been conclusively proven in a lot of studies [29,31,32]. In the current study, we found the sheep with TT genotype at SNP7 in 3′UTR had a higher mean value of body weight, withers height, and chest circumference than CT and CC genotypes. From the routine perceiving, the mutant T at 10,044 bp of *MYF5* might be a beneficial allele for muscle development in terms of appearance. Whether this mutation would be favorable to muscle growth without other side effects needs more investigation.

Among the seven SNPs found in the present study, the SNP1, SNP2, and SNP3 are located in the promoter region of ovine *MYF5*. The luciferase assay determined that SNP2 interacted with transcription factor SP1, SNP1 was significantly associated with the body weight and measurement traits, and SNP3 was significantly associated with eye muscle area. A promoter contains important information about the network of the gene expression regulation [33], and single nucleotide polymorphisms in the promoter regions can affect the gene’s regulation activity by altering the binding efficiency of the transcript factors [34,35]. For instance, Kostek et al. reported that a polymorphism in the *IGF1* promoter is significantly associated with total human fat mass [36]. From our study, the results of related SNPs in *MYF5* promoter suggest that three SNPs may affect the transcriptional efficiency by changing its promoter regulating activity, and SNP2 specifically may affect the SP1 binding efficiency to regulate the promoter’s activity. Of course, more precise assays such as immunoprecipitation need to be conducted to ensure the interaction between SP1 and the binding site containing SNP2.

## 5. Conclusions

This is the first report describing the variation of ovine *MYF5* in its whole length, and the association between variations and body weight, and body measurements and ultrasound traits in sheep. Through the investigation, seven SNPs were identified in a Chinese indigenous sheep breed and four of them were novel in identification. In addition, g.6838G > A, g.7117 C > A, g.8371 T > C, g.9471 T > G, and g.10044 C > T and diplotypes of H1H3 and H2H3 in GSTS might influence body weight, body measurements, and *longissimus dosi* ultrasound traits, and haplotype of H3 would be beneficial for improving meat production. These findings laid the foundation for formulating the breeding regime for grassland small-tailed sheep and provided a deep insight into offering a functional genetic marker for improving economically valuable traits in sheep breeding.

## Figures and Tables

**Table 1:** Genetic parameters, genotypic, and allelic frequencies of *MYF5*.

ID	Locus	Position	Genotypic Frequency ^$^			Allele Frequency		χ^2^ (HWE)	ObsHe	PredHe	PIC
SNP1	g.6838G > A	Promoter	GG 400/0.750	GA 123/0.231	AA 10/0.019	G 0.866	A 0.134	*p* > 0.05	0.230	0.232	0.205
SNP2	g.6989G > T	Promoter	GG 96/0.180	GT 237/0.445	TT 200/0.375	G 0.402	T 0.598	*p* > 0.05	0.444	0.481	0.365
SNP3	g.7106C > A	Promoter	CC 59/0.111	CA 230/0.432	AA 244/0.457	C 0.326	A 0.674	*p* > 0.05	0.431	0.440	0.343
SNP4	g.8371T > C	Intron1	TT 351/0.659	CT 166/0.311	CC 16/0.030	T 0.814	C 0.186	*p* > 0.05	0.311	0.302	0.257
SNP5	g.8561G > A	Intron1	GG 383/0.719	GA 140/0.263	AA 10/0.019	G 0.850	A 0.150	*p* > 0.05	0.262	0.255	0.223
SNP6	g.9471T > G	Intron2	TT 376/0.705	GT 143/0.268	GG 14/0.026	T 0.840	G 0.160	*p* > 0.05	0.266	0.268	0.233
SNP7	g.10044C > T	3′UTR	CC 366/0.687	CT 156/0.293	TT 11/0.066	C 0.833	T 0.167	*p* > 0.05	0.292	0.278	0.240

^$^ The number before the slash represents the quantity of individuals and after it the frequency; HWE, Hardy–Weinberg equilibrium; ObsHe, observation heterozygosity; PredHe, prediction heterozygosity; PIC, polymorphism information content; 3′UTR, 3′ untranslated region.

**Table 2:** Haplotypes of *MYF5* gene and the frequencies in GSTS flock.

Haplotype	SNP1	SNP2	SNP3	SNP4	SNP5	SNP6	SNP7	Frequency
	g.6838G > A	g.6989G > T	g.7106C > A	g.8371T > C	g.8561G > A	g.9471T > G	g.10044C > T	
H1	G	T	A	T	G	T	C	0.497
H2	G	G	C	T	G	T	C	0.222
H3	A	G	A	C	A	G	T	0.126
H4	G	T	A	C	G	T	C	0.038
H5	G	T	C	T	G	T	C	0.036
H6	G	G	C	T	G	T	T	0.025
H7	G	G	C	T	G	G	C	0.019
H8	G	T	C	C	A	T	C	0.017

**Table 3:** Diplotypes of *MYF5* gene and the frequencies in GSTS flock.

Diplotypes	SNP1	SNP2	SNP3	SNP4	SNP5	SNP6	SNP7	Sample Size	Frequency
	g.6838G > A	g.6989G > T	g.7106C > A	g.8371T > C	g.8561G > A	g.9471T > G	g.10044C > T		
H1H1	G G	T T	A A	T T	G G	T T	C C	144	0.256
H1H2	G G	T G	A C	T T	G G	T T	C C	128	0.224
H1H3	G A	T G	A A	T C	G A	T G	C T	59	0.105
H2H3	G A	G G	C A	T C	G A	T G	C T	42	0.075

**Table 4:** Association between mutations in *MYF5* with body measurements and ultrasound traits.

Locus	Genotype	BW (kg)	BL (cm)	WH (cm)	CD (cm)	CC (cm)	CW (cm)	CBC (cm)	HW (cm)	EMA (mm^2^)	BFT (mm)
SNP1	AA(10)	55.88 ± 15.75 ^a^	72.10 ± 5.23	76.80 ± 5.75 ^a^	40.60 ± 2.63	98.6 ± 10.05 ^a^	20.40 ± 1.71 ^a^	10.15 ± 0.94 ^a^	24.00 ± 2.49	1040.04 ± 376.83	5.29 ± 2.36
	GA(122)	51.85 ± 9.59 ^b^	73.36 ± 5.42	75.31 ± 4.16 ^b^	40.85 ± 2.08	96.09 ± 6.52 ^b^	19.85 ± 1.50 ^a,b^	9.84 ± 0.80 ^b^	23.31 ± 1.95	1030.53 ± 294.14	4.61 ± 1.62
	GG(400)	50.34 ± 9.88 ^b^	72.12 ± 5.43	74.52 ± 4.01 ^b^	40.42 ± 2.23	95.33 ± 6.85 ^b^	19.51 ± 1.67 ^b^	9.77 ± 0.75 ^b^	23.12 ± 1.96	995.38 ± 281.72	4.50 ± 1.57
SNP2	GG(96)	51.13 ± 10.28	72.69 ± 5.08	74.82 ± 3.72	40.67 ± 2.09	95.42 ± 6.91	19.59 ± 1.47	9.79 ± 0.80	23.06 ± 1.87	979.20 ± 293.43	4.56 ± 1.65
	GT(236)	50.66 ± 10.09	72.49 ± 5.60	74.81 ± 4.16	40.51 ± 2.36	95.65 ± 6.87	19.61 ± 1.69	9.76 ± 0.80	23.17 ± 2.01	1020.04 ± 308.51	4.55 ± 1.61
	TT(200)	50.78 ± 9.73	72.16 ± 5.43	74.62 ± 4.20	40.47 ± 2.08	95.54 ± 6.83	19.60 ± 1.66	9.83 ± 0.72	23.26 ± 1.97	997.74 ± 254.15	4.51 ± 1.57
SNP3	CC(59)	50.23 ± 10.49	72.64 ± 5.92	74.49 ± 3.62	40.45 ± 2.31	95.13 ± 6.75	19.37 ± 1.51	9.73 ± 0.72	22.98 ± 1.72	953.17 ± 279.64 ^b^	4.46 ± 1.69
	AC(229)	51.02 ± 9.92	72.53 ± 5.34	74.84 ± 4.14	40.59 ± 2.24	95.48 ± 6.77	19.58 ± 1.62	9.75 ± 0.82	23.16 ± 1.99	1018.16 ± 310.42 ^a^	4.54 ± 1.59
	AA(244)	50.71 ± 9.93	72.22 ± 5.43	74.71 ± 4.17	40.48 ± 2.16	95.75 ± 6.96	19.68 ± 1.68	9.85 ± 0.72	23.25 ± 2.01	1003.65 ± 263.16 ^a,b^	4.55 ± 1.60
SNP4	CC(16)	47.17 ± 3.92 ^b^	70.56 ± 2.7 ^b^	74.00 ± 1.67 ^b^	40.56 ± 2.42	93.43 ± 4.41 ^b^	19.68 ± 1.30	9.46 ± 0.38 ^b^	22.62 ± 1.40	874.80 ± 158.68 ^b^	4.13 ± 0.87
	CT(166)	52.23 ± 10.0 4 ^a^	73.19 ± 5.3 2 ^a^	75.54 ± 4.41 ^a^	40.92 ± 2.14	96.16 ± 6.92 ^a^	19.81 ± 1.57	9.89 ± 0.82 ^a^	23.35 ± 1.98	1036.16 ± 291.39 ^a^	4.65 ± 1.66
	TT(351)	50.29 ± 10.0 4 ^a^	72.11 ± 5.55 ^a,b^	74.40 ± 3.96 ^b^	40.33 ± 2.21	95.38 ± 6.88 ^ab^	19.50 ± 1.67	9.76 ± 0.75 ^a^	23.13 ± 1.98	994.65 ± 286.43 ^a^	4.50 ± 1.60
SNP5	AA(10)	53.33 ± 14.99	71.20 ± 5.07	75.50 ± 4.83	40.40 ± 2.50	97.00 ± 9.09	20.50 ± 1.64 ^a^	10.00 ± 0.91	23.70 ± 2.40	976.52 ± 349.50	4.55 ± 1.60
	GA(140)	52.01 ± 9.55	73.21 ± 5.30	75.52 ± 4.36	40.99 ± 2.15	96.03 ± 6.63	19.79 ± 1.52 ^a,b^	9.84 ± 0.81	23.30 ± 1.95	1034.40 ± 287.75	4.68 ± 1.66
	GG(383)	50.29 ± 9.94	72.13 ± 5.48	74.44 ± 3.94	40.36 ± 2.20	95.36 ± 6.86	19.51 ± 1.67 ^b^	9.77 ± 0.75	23.13 ± 1.96	993.57 ± 283.87	4.48 ± 1.58
SNP6	GG(14)	53.60 ± 14.46 ^a^	71.78 ± 4.91	75.92 ± 5.34 ^a^	40.64 ± 2.27	97.35 ± 9.42	19.85 ± 1.74	10.17 ± 0.82 ^a^	23.28 ± 2.46	1046.58 ± 330.02	4.75 ± 2.14
	GT(142)	52.04 ± 9.60 ^a,b^	73.26 ± 5.42	75.37 ± 4.05 ^a,b^	40.89 ± 2.19	96.09 ± 6.67	19.82 ± 1.55	9.85 ± 0.79 ^b^	23.34 ± 1.95	1025.65 ± 291.46	4.74 ± 1.67
	TT(376)	50.26 ± 9.86 ^b^	72.11 ± 5.44	74.47 ± 4.03 ^b^	40.39 ± 2.20	95.31 ± 6.81	19.51 ± 1.66	9.76 ± 0.75 ^b^	23.12 ± 1.96	994.31 ± 283.06	4.45 ± 1.55
SNP7	TT(11)	54.97 ± 15.25 ^a^	72.27 ± 5.00	76.63 ± 5.48 ^a^	40.63 ± 2.50	98.00 ± 9.74 ^a^	20.18 ± 1.77	10.04 ± 0.96	23.63 ± 2.65	1037.14 ± 357.62	5.04 ± 2.39
	CT(156)	51.20 ± 9.58 ^b^	72.96 ± 5.46	75.07 ± 4.03 ^b^	40.68 ± 2.19	95.62 ± 6.59 ^a,b^	19.72 ± 1.53	9.82 ± 0.79	23.19 ± 1.87	1009.6 ± 290.99	4.60 ± 1.62
	CC(366)	50.50 ± 9.93 ^b^	72.16 ± 5.43	74.54 ± 4.06 ^b^	40.45 ± 2.21	95.47 ± 6.86 ^b^	19.53 ± 1.67	9.77 ± 0.75	23.16 ± 1.99	1000.6 ± 282.57	4.49 ± 1.57

Values are shown as the least squares means ± standard deviation; ^a,b^ Means with different superscripts are significantly different (*p* < 0.05); body weight (BW), body length (BL), withers height (WH), chest depth (CD), chest circumference (CC), chest width (CW), cannon bone circumference (CBC), hip width (HW). Ultrasound traits including eye muscle area (EMA) and backfat thickness (BFT).

**Table 5:** Associations of combined genotypes with body measurements and ultrasound traits in GSTS flock.

Combined Genotypes	BW (kg)	BL (cm)	WH (cm)	CD (cm)	CC (cm)	CW (cm)	CBC (cm)	HW (cm)	EMA (mm^2^)	BFT (mm)
H1H1(144)	49.90 ± 9.48 ^b^	71.61 ± 5.21 ^b^	74.20 ± 3.92 ^b^	40.29 ± 2.01	95.18 ± 6.91 ^b^	19.43 ± 1.72 ^b^	9.79 ± 0.68 ^a,b^	23.10 ± 2.02 ^b^	986.39 ± 253.01	4.43 ± 1.48
H1H2(128)	49.60 ± 9.40 ^b^	71.77 ± 5.19 ^b^	74.12 ± 3.79 ^b^	40.25 ± 2.32	95.00 ± 6.58 ^b^	19.45 ± 1.72 ^b^	9.69 ± 0.77 ^b^	23.00 ± 1.97 ^b^	1006.77 ± 317.83	4.50 ± 1.59
H1H3(59)	52.49 ± 10.23 ^a^	73.67 ± 6.06 ^a^	75.72 ± 4.65 ^a^	40.91 ± 2.29	97.01 ± 6.86 ^a^	20.16 ± 1.60 ^a^	9.93 ± 0.80 ^a^	23.72 ± 2.05 ^a^	1047.53 ± 283.50	4.84 ± 1.77
H2H3(42)	51.90 ± 8.25 ^a^	73.54 ± 4.55 ^a^	75.02 ± 3.47 ^a^	40.88 ± 1.83	95.50 ± 6.20 ^a,b^	19.69 ± 1.35 ^a,b^	9.76 ± 0.82 ^a,b^	23.11 ± 1.72 ^b^	1033.34 ± 289.56	4.57 ± 1.49

a,b Means with different super-scripts are significantly different (*p* < 0.05).

**Table 6:** The effect of haplotype H3 to the body measurements traits and ultrasound traits in GSTS flock.

Combined Genotypes	BW (kg)	BL (cm)	WH (cm)	CD (cm)	CC (cm)	CW (cm)	CBC (cm)	HW (cm)	EMA (mm2)	BFT (mm)
H3+(131)	52.29 ± 10.22	73.26 ± 5.46	75.47 ± 4.32	40.85 ± 2.12	96.34 ± 6.89	19.89 ± 1.54	9.87 ± 0.81	23.37 ± 2.01	1037.31 ± 300.13	4.69 ± 1.68
H3-(402)	50.27 ± 9.8	72.14 ± 5.41	74.52 ± 3.99	40.43 ± 2.22	95.33 ± 6.83	19.5 ± 1.66	9.77 ± 0.75	23.12 ± 1.96	993.77 ± 281.72	4.49 ± 1.57
*p*	0.044	0.042	0.022	0.058	0.142	0.019	0.169	0.206	0.133	0.208

## Data Availability

The datasets used and analyzed during the current study are available from the corresponding author on reasonable request.

## Supplementary Materials

The following supporting information can be downloaded at: https://www.mdpi.com/article/10.3390/genes13030483/s1, Table S1: Primers and amplification conditions for identifying SNPs; Table S2: SNaPshot single base extension primers; Table S3: Homologous recombination primer designed for construction of expression vector; Table S4: The primer information of the base mutation of SNP2.
