# Supplementary material for: Novel Single Nucleotide Polymorphisms and Haplotype of MYF5 Gene Are Associated with Body Measurements and Ultrasound Traits in Grassland Short-Tailed Sheep
Source: Genes (Basel). 2022 Mar 9;13(3):483. doi: 10.3390/genes13030483 (PMC8949509; doi:10.3390/genes13030483)
Supplement: Supplementary file 1 [file genes-13-00483-s001.zip › genes-1614583-supplementary.pdf]

Table S1. Primers and amplification conditions for identifying SNPs.

Name	Primer (5′-3′)	Tm(℃)	Production size (bp)	Amplified region
MYF5-1	F:AGGAGCCAGAT-TTTCAAGGG<br>R:CCACAG-TCTAAACAGCCAAGC	61	792	Promoter
MYF5-2	F:ACACGAGCCAAGAAA-GAT<br>R:TTACCTAAAC-CAAGGGACA	57	771	Promoter
MYF5-3	F:GCAAGTAAGCG-GATTGGG<br>R:TGGTTAGGGTTGGTCGTG	61	729	5′UTR, partial exon1
MYF5-4	F:GAGTGGCTGCTTTCGG<br>R:GTTGCCTTTA-GATGTTAGTGTATG	57	758	Partial exon1, partial intron1
MYF5-5	F:GGATAGTTGGG-TATTGGG<br>R:GA-TAGGGCTGTTACATTCA	57	786	Intron1, partial exon2
MYF5-6	F:CCTTGCGATTGGTGAG<br>R:TGATAAATGAGCCTG-GAA	60	824	Exon2, intron2, exon3
MYF5-7	F:CAGCACCGATTCTCAG<br>R:ATACCAACAGGACCTAA	60	823	3′UTR

Table S2. SNaPshot single base extension primers

Name	Primer（5′-3′）	Production size	Amplified region
MYF5-P1	F:CCTGAGCTCGCTAGCCTCGAGTGGTGGTGGTAGAGGTTGTGTCT	1859bp	-1799/+59
MYF5-P2	F:CCTGAGCTCGCTAGCCTCGAGAGTGTGTGAAGTTACTCGGTCG	1257bp	-1197/+59
MYF5-P3	F:CCTGAGCTCGCTAGCCTCGAGAAAGAGAGAAAGCCAGGGGTG	926bp	-866/+59
MYF5-P4	F:CCTGAGCTCGCTAGCCTCGAGGGTGTCAACTGAAATGGGGAGC	574bp	-514/+59
MYF5-P5	F:CCTGAGCTCGCTAGCCTCGAGCGGTGGGATATGCTAATAGTGCG	301bp	-241/+59
MYF5-R	R:CAGTACCGGATTGCCAAGCTTCAGGAGCCGTCGTAGAAGTACTC		

Table S3. Homologous recombination primer designed for construction of expression vector

SNP name	Primer (5′-3′)	Tm(℃)
SNP1	TTTTTTTTTTTAGAGGGAGAAGGGAGACGA	55
SNP2	TTTTTTTTTTTTTGGGAAAGAGAGAAAGCCAGG	55
SNP3	TTTTTTTTTTTTTTTTCCTCAATAACACAAAGCCTACATA	55
SNP4	TTTTTTTTTTTTTTTTTTTTTTTTTCTCTGTACCTGCTAGGGCTT	58
SNP5	TTTTTTTTTTTTTTTTTTTTTTTTTTTTCCTACTCTAGGTGCACACTGAA	58
SNP6	TTTTTTTTTTTTTTTTTTTTTTTTTTTTTTTTTTTTGTATGTCAAGGTCCACTGG	58
SNP7	TATATCTAAAGCAAAGATGAAGGAAAA	60

Table S4 The primer information of the base mutation of SNP2

Primer name	Sequence (5′-3′)	Tm(℃)
Base mutation F	CACAAAGCCTACATAATGGCAATCTG	55
Base mutation R	GGGAGTTATTGTGTTTCGGATGTATT	
Wild type F	CTCGAGTCAACAAACAGGCGCAGAA	55
Wild type R	AAGCTTACAGGAGGGCCAGGTGACCA	
